# Greater confidence in management of food allergy by parents predicts better quality of life

**DOI:** 10.1186/2045-7022-5-S3-O7

**Published:** 2015-03-30

**Authors:** Rebecca Knibb, Christopher Barnes, Carol Stalker

**Affiliations:** 1 Aston University, Birmingham, UK; 2 University of Derby, Derby, UK

## 

Food allergy is often a life-long condition and has been shown to have a significant impact on the quality of life of the patient and parents due to the complexity of managing food allergy to avoid potentially life threatening reactions (Cummings et al., 2010). Parental confidence or self-efficacy in managing food allergy for their child has not been investigated in this population. This study aimed to examine whether self-efficacy in parents of food allergic children was a good predictor of quality of life of the family.

Parents of children with clinically diagnosed food allergy (N=434) completed a food allergy demographic questionnaire, the Food Allergy Self-Efficacy Scale for Parents (FASE-P), the Food Allergy Quality of Life Parental Burden Scale (FAQL-PB), the GHQ-12 (to measure mental health) and the Food Allergy Independent Measure (FAIM), which measures perceived likelihood of a severe allergic reaction.

Greater quality of life as measured by the FAQL-PB was significantly related to greater self-efficacy for food allergy management, better mental health, lower perceived likelihood of a severe reaction, older age in parent and child and fewer number of allergies (p<0.05). Significantly poorer quality of life was reported in parents of children who had asthma, eczema, egg allergy, history of anaphylaxis or hospitalisation due to food allergy (p<0.05). Food allergy self-efficacy, food allergy severity, mental health, age of child and number of allergies significantly predicted quality of life with self-efficacy being the biggest predictor (p<0.01, adj R^2^=.46).

Confidence in being able to manage your child’s food allergy is important and is associated with better parental quality of life, in addition to parental mental health and food allergy characteristics. Interventions to improve self-efficacy in parents of food allergic children should be explored.

